# Quality of life among district hospital nurses with multisite musculoskeletal symptoms in Vietnam

**DOI:** 10.1002/1348-9585.12161

**Published:** 2020-09-19

**Authors:** Thanh Hai Nguyen, Duc Luan Hoang, Thi Giang Hoang, Minh Khue Pham, Van Khai Nguyen, Julie Bodin, Jean‐Dominique Dewitte, Yves Roquelaure

**Affiliations:** ^1^ Faculty of Public Health Haiphong University of Medicine and Pharmacy Haiphong Vietnam; ^2^ Univ Angers CHU Angers Univ Rennes Inserm EHESP, Irset (Institut de recherche en santé, environnement et travail) ‐ UMR_S 1085 Angers France; ^3^ Phu Tho College of Medicine and Pharmacy Phu Tho Vietnam; ^4^ Occupational Health and Environmental Diseases Department CHRU Morvan ‐ Laboratory for Studies and Research in Sociology (EA3149) University of Western Brittany Brest France

**Keywords:** district hospital, musculoskeletal disorders, nurses, quality of life, Vietnam

## Abstract

**Background:**

Nurses are one of the population groups with the highest prevalence of musculoskeletal disorders (MSDs). At many sites, musculoskeletal symptoms (MS) represent a major health‐care burden, adversely affecting nurses' quality of life and giving rise to mental health issues.

**Objectives:**

This study measured the prevalence of multi‐body‐site (two or more anatomical sites) musculoskeletal symptoms (MMS), and the association between MMS, a number of demographic and work characteristics, psychological distress, and the quality of life among district hospital nurses.

**Material and Methods:**

A cross‐sectional study was performed with 1179 nurses in Haiphong City using three questionnaires: the Modified Nordic; Quality of Life Enjoyment and Satisfaction Short Form (Q‐LES‐Q‐SF); and the Kessler Psychological Distress Questionnaire (K6).

**Results:**

Women have a higher MMS prevalence than men (57.1% in women vs 37.6% in men, *P* < .001). Having a higher number of anatomical sites of MS appears to be associated with a worse quality of life among nurses. Linear regression analysis found a number of other factors negatively associated with the nurses' quality of life: gender (female), age (50‐60 years old vs 19‐29 years old), and psychological distress.

**Conclusions:**

This study shows a high prevalence of MMS and the relationship between, on the one hand, MMS, gender, age, as well as psychological distress and, on the other hand, the quality of life among nurses in Vietnam. Further in‐depth studies are needed to investigate the causal relationships between these indicators.

## 1 INTRODUCTION

Musculoskeletal disorders (MSDs) continue to be the most prevalent occupational health problem among workers, representing 60% of all self‐reported problems across the 28 European Union Member States. Among these conditions, backache accounted for 43%, followed by muscular pains in the neck or upper limbs (42%), and muscular pains in the hip or lower limbs (29%).^1^ Workers in all sectors and occupations can be affected. Health professionals are no exception, with the reported prevalence ranging from 28% to 96% during a period of one year.^2^ Nurses constitute one of the groups with the highest prevalence of MSDs. A systematic review estimated an average of musculoskeletal symptoms (MS) in nurses at 71.85%.^3^

Nurses play an important role in the provision of health‐care services to patients. Factors that affect the nurses' health, as well as their quality of life and work‐life balance, are expected to affect the quality of health‐care services provided by them and their patients' level of satisfaction.^4^ MSDs in nurses are also a major health‐care burden because of working days lost due to sick leave, diminished productivity, and a decline in the quality of patient care.^5^

Studies on nurses' MSDs have only considered the relation between determinants and simple MSDs (in each anatomical site), but not multisite musculoskeletal symptoms (MMS). There are very few studies of MMS on health‐care workers in general or on various subpopulations with a high prevalence of MMS, such as nurses.^6^, ^7^ This situation is similar for both the general population and for the working population.^8^

Although quality of life has been subject to extensive research in the general population as well as in patients with various diseases, there are a limited number of studies that consider specific occupational categories, such as nurses. Konstantinou et al showed that the quality of life scores of nurses in general fell in the middle of the scale.^9^ Our question is: how do MMS further affect the quality of life for nurses?

The relationship between mental health and MMS among medical staff (including nurses) has been documented in various studies,^6^, ^10^ and although some have noted the association between the three elements of MMS, quality of life, and mental health,^11^ their focus has not been on nurses. In Vietnam, several studies have shown a high prevalence of MSDs in general, and also a high prevalence of MMS in hospital nurses during a one‐year period.^12^, ^13^ However, the effects of MSDs, in particular MMS, and a number of demographic and work characteristics, as well as psychological distress on the quality of life of Vietnamese nurses, have not been studied and evaluated. The absence of such work partly informs the rationale behind this study.

Haiphong is one of the largest cities in Vietnam with more than two million inhabitants and acts as the main seaport for the northern region of the country. According to the Vietnamese Health Statistics Yearbook in 2016, Haiphong has a total of 281 medical establishments, including 15 public district hospitals, with a ratio of 40 beds and 7.7 doctors per 10 000 inhabitants.^14^ The main function of public district hospitals is to receive all cases of patients coming from outside or from lower‐level health facilities transferred for emergency, inpatient, or outpatient medical examination and treatment.

The objective of this study is to measure the prevalence of MMS and the association between MMS, a number of demographic and work characteristics, psychological distress, and the quality of life among district hospital nurses in Haiphong.

## 2 MATERIALS AND METHODS

A descriptive cross‐sectional survey was performed between January and June 2017 to ascertain the quality of life, MMS, psychological distress, and some potential explanatory variables among nurses working in the public district hospitals of Haiphong, Vietnam.

### 2.1 Eligibility criteria

We selected all nurses working in the 15 public district hospitals of Haiphong who meet the following criteria:

1. Have a nursing diploma (diplomas at different levels such as university, college, secondary or elementary, etc);
2. Have worked in the hospital for at least 9 months immediately prior to the start of the study (the number of nurses working in the hospital from 9 to 12 months was also important; in order to have a larger representative sample, all such nurses were selected);
3. Had no persistent musculoskeletal system problems or diseases or preexisting pathologies such as congenital spinal disorders, trauma and pain from surgery, or other trauma diseases, etc; and
4. Agree to participate in the study.

A total of 1179/1279 nurses participated in the study.

### 2.2 Research instruments

Before the research began, ten interviewers were trained with the questionnaires. In parallel with this, communication with hospital leaders was conducted to gain consent and to schedule nursing interviews for each hospital. After arranging the appointments, the interviewers visited the hospital to interview the nurses. The nurses were summoned one at a time in order to lessen the impact on their work. The questionnaires were used by our researcher for direct interviews. These ranged from 30 to 45 minutes in length.

Four questionnaires were used for the interview:

1. A sociodemographic questionnaire: This was used to collect general information, such as age, gender, height, weight, personal history of musculoskeletal diseases across their whole life, and work‐related information, including number of working hours (per day, per week), seniority, and working patterns (shift work).
2. A Modified Nordic Questionnaire: This was based on the Standardized Nordic Questionnaire developed by Kuorinka et al in 1987.^15^ This questionnaire was translated into Vietnamese and tested on a small group of subjects. Before use in the study, it was slightly modified to fit the Vietnamese context. This questionnaire evaluates health problems of the musculoskeletal system at nine different positions on the body (neck, shoulder/upper arm, elbow/forearm, wrist/hand, upper back, lower back, hip/thigh, knee/lower leg, and ankle/foot) during the past 12 months, and the impact of those problems on the work and life of the respondent.
3. A Quality of Life Enjoyment and Satisfaction Questionnaire Short Form (Q‐LES‐Q‐SF): This was developed by Stevanovic et al^16^ from an original longform quality of life enjoyment and satisfaction questionnaire (Q‐LES‐Q) developed by Endicott et al in 1993. The short form, with 16 items, evaluates overall enjoyment and satisfaction with physical health, mood, work, household and leisure activities, social and family relationships, daily functioning, sexual life, economic status, overall wellbeing, and medications. Items are rated on a five‐point scale (“not at all or never” to “frequently or all the time”). The scoring of the Q‐LES‐Q‐SF involves totaling the first 14 items only to yield a total score (ranges from 14 to 70) with higher scores indicating better quality of life. The last two items, which deal with medications and overall life satisfaction, are not included in the total score. This is the reason we used only 14 items for the interviews. This short form was validated in the Vietnamese language by To Gia Kien et al in 2013.^17^
4. The Kessler Psychological Distress Scale (K6): This short questionnaire consists of six questions about a person's emotional state (nervous, hopeless, restless or fidgety, so depressed that nothing can cheer you up, everything is an effort, and worthless).^18^ Each question is scored from 0 to 4 (corresponding to "None of the time" through to "All of the time"). The total score, was obtained by calculating the score from the six questions, with totals ranging from 0 to 24. A higher score indicates a more serious level of psychological distress.

### 2.3 The explanatory variables studied

These variables were used to describe populations and were included in the multivariate linear regression analysis:

1. Musculoskeletal symptoms (MS) among the nine anatomical sites studied: no symptom at any site, symptom at one anatomical site, symptom at two to four anatomical sites, and symptom at five sites or more (MMS is defined as MS at two or more anatomical sites)
2. Psychological distress
3. Gender: men/women
4. Age group: 19‐29, 30‐39, 40‐49, and 50‐60 years old
5. Body mass index (BMI): < 18.5 (underweight), 18.5‐24.9 (normal), and ≥25 (overweight)
6. Seniority: less than five years, five to ten years, ten to fifteen years, and more than fifteen years
7. Number of working hours per week: ≤50 and >50 hours
8. Shift work: yes/no
9. History of musculoskeletal diseases: yes/no
10. Location of the hospital: rural or urban region

### 2.4 Missing data treatment

The face to face interview method used in this study has minimized data missing as much as possible. In fact, most of the research data were complete. In case of missing data detected during the review of the questionnaire, our researcher contacted the respondent with the code on the form to retrieve the missing information. Such procedures conduct a complete‐case for data analysis.

### 2.5 Statistical analysis

SPSS version 22.0 software was used for data analysis. Prevalence (number, percentage) of MS in each category according to sociodemographic and occupational variables was calculated. A chi‐square test for qualitative variables and student t‐tests or ANOVA test for continuous variables were used to compare characteristics of the sample according to MS. Linear regression analysis was used to study the relationship between quality of life and MMS. All other potential explanatory variables have been treated as confounders. Two analytical steps were conducted: the first step involved linear regression with each of the independent variables adjusted for gender; and in the second step, variables with *P* value less than .2 in step 1 and inviolate multi‐collinearity were included in a multivariate regression analysis.

## 3 RESULTS

### 3.1 Some sociodemographic characteristics of participants

Among the nurses participating in the study, 81.3% (958/1179) were female. The mean age was 32.6 years (SD = 7.7) and over 80% of the study group was under 40 years old. More than 80% of the nurses had a BMI index in the normal range (984/1179). Around 34% had a seniority of less than five years or between five and ten years. The number of nurses working regularly for more than 50 hours per week was 38.6%, and more than two thirds (68.3%) were on shift work. The percentage of nurses with a history of musculoskeletal diseases was 11.2%, and more than two thirds of nurses worked in hospitals located in rural areas (Table 1).

**TABLE 1:** Characteristics of participants according to musculoskeletal symptom category

Characteristics	N = 1179	No site	MS at 1 site	MMS		*P*‐value
				MS at 2 to 4 sites	MS at 5 sites or more	
	n	n (%)				
Gender						<.001
Men	221	87 (39.4)	51 (23.1)	68 (30.8)	15 (6.8)	
Women	958	215 (22.4)	196 (20.5)	427 (44.6)	120 (12.5)	
Age group (y) (mean ± standard deviation = 32.6 ± 7.7)						<.001
19‐29	477	139 (29.1)	106 (22.2)	191 (40.0)	41 (8.6)	
30‐39	487	113 (23.2)	99 (20.3)	227 (46.6)	48 (9.9)	
40‐49	166	39 (23.5)	31 (18.7)	65 (39.2)	31 (18.7)	
50‐60	49	11 (22.4)	11 (22.4)	12 (24.5)	15 (30.6)	
BMI (mean ± standard deviation = 21.0 ± 2.2)						.854
<18.5 underweight	134	31 (23.1)	27 (20.1)	60 (44.8)	16 (11.9)	
18.5‐24.9 normal	984	251 (25.5)	208 (21.1)	411 (41.8)	114 (11.6)	
≥25 overweight	61	20 (32.8)	12 (19.7)	24 (39.3)	5 (8.2)	
Seniority						<.001
Less than 5 y	408	122 (29.9)	92 (22.5)	161 (39.5)	33 (8.1)	
5‐10 y	405	103 (25.4)	84 (20.7)	183 (45.2)	35 (8.6)	
10‐15 y	183	38 (20.8)	32 (17.5)	91 (49.7)	22 (12.0)	
More than 15 y	183	39 (21.3)	39 (21.3)	60 (32.8)	45 (24.6)	
Number of working hours per week						.531
≤50 h	724	182 (25.1)	159 (22.0)	306 (42.3)	77 (10.6)	
>50 h	455	120 (26.4)	88 (19.3)	189 (41.5)	58 (12.7)	
Shift work						.09
No	374	84 (22.5)	88 (23.5)	151 (40.4)	51 (13.6)	
Yes	805	218 (27.1)	159 (19.8)	344 (42.7)	84 (10.4)	
History of musculoskeletal diseases						<.001
No	1047	294 (28.1)	224 (21.4)	425 (40.6)	104 (9.9)	
Yes	132	8 (6.1)	23 (17.4)	70 (53.0)	31 (23.5)	
Location of hospital						.012
Rural region	798	217 (27.2)	177 (22.2)	326 (40.9)	78 (9.8)	
Urban region	381	85 (22.3)	70 (18.4)	169 (44.4)	57 (15.0)	

	M ± SD				
Quality of life	53.2 ± 7.2	52.0 ± 6.1	49.5 ± 6.3	46.9 ± 6.1	<.001
Psychological distress	3.5 ± 3.3	3.5 ± 3.4	5.2 ± 3.6	6.3 ± 4.0	<.001

Abbreviations: MMS, Multisite musculoskeletal symptoms (two or more anatomical sites); MS, Musculoskeletal symptoms (at least one anatomical site).

### 3.2 Prevalence of multisite musculoskeletal symptoms

This study's results are the first to show that women have significantly higher MMS prevalence than men: 57.1% (95% CI: 50.6%‐63.6%) in women compared with 37.6% (95% CI: 34.5%‐40.7%) in men, *P* < .001. Among those who reported having MS in at least one of any nine anatomical sites, the number of nurses reporting symptoms at two to four anatomical sites accounted for the highest percentage. In the category of MS at two to four sites, the highest percentage (46.6%, 95% CI: 42.2%‐51.0%) belonged to the 30‐39 age group, while the 50‐60 age group had the highest proportion in the category of MS at five sites or more (*P* < .001). In terms of seniority, the highest percentage in the category of MS at one site, two to four sites, and five sites or more were less than 5 years group (22.5%, 95% CI: 18.4%‐26.6%), 10‐15 years group (49.7%, 95% CI: 42.5%‐56.9%), and more than 15 years group (24.6%, 95% CI: 18.4%‐30.8%) respectively (*P* < .001). Nurses with personal history of musculoskeletal diseases had a higher proportion of symptoms than those without this history in the category of MS at two to four sites (53.0%, 95% CI: 44.5%‐61.5%, vs 40.6%, 95% CI: 37.6%‐43.6%, respectively) and at five sites or more (23.5%, 95% CI: 16.3%‐30.7%, compared to 9.9%, 95% CI: 8.1%‐11.7%, respectively) (*P* < .001). Nurses working in hospitals located in urban regions had a higher percentage of MS at two to four sites and five sites or more than those in rural regions (44.4%, 95% CI: 39.4%‐49.4%, vs 40.9%, 95% CI: 37.5%‐44.3%; and 15.0%, 95% CI: 11.4%‐18.6%, vs 9.8%, 95% CI: 7.7%‐11.9%, respectively with *P* = .012). The difference in MS prevalence according to BMI, the number of working hours per week, and shift work were shown with *P* values of .854, .531, and .09 respectively.

### 3.3 Scores of Q‐LES‐Q‐SF and psychological distress

According to MS category, the average score for the quality of life of nurses decreased from the non‐MS group to the group of MS at five sites or more: 53.2 (SD = 7.2), 52.0 (SD = 6.1), 49.5 (SD = 6.3), and 46.9 (SD = 6.1) respectively. In contrast, the average score of psychological distress increased with the increase in the number of MS: 3.5 with non‐MS group (SD = 3.3) and the group of MS at one site (SD = 3.4), 5.2 (SD = 3.6) in MS at two to four sites, and 6.3 (SD = 4.0) with the group of MS at five sites or more (Table 1).

### 3.4 Linear regression of quality of life enjoyment and satisfaction

Table 2 summarizes the results of linear regressions of the quality of life with each potential factor (including MMS and psychological distress) after adjustment for gender. Most independent variables had a *P* value of less than .2, except for BMI and work more than 50 hours per week.

**TABLE 2:** Linear regression analysis of quality of life with each of the potential explanatory variables adjusted for gender

Independent variable	β^a^	95% CI	*P*
Musculoskeletal symptoms (reference: no site)			<.001
1 site	−1.02	−2.10; 0.07	
2‐4 sites	−3.38	−4.31; −2.45	
5 sites or more	−5.89	−7.21; −4.58	
Female gender (reference: male gender)	−3.02	−4.00; −2.04	<.001
Age (reference: 19‐29)			.081
30‐39	0.59	−0.27; 1.44	
40‐49	−0.17	−1.36; 1.02	
50‐60	−1.81	−3.78; 0.17	
BMI (reference: normal [18.5‐24.9 kg/m^2^])			.353
Underweight (<18.5 kg/m^2^)	−0.89	−2.10; 0.32	
Overweight or obesity (≥25 kg/m^2^)	−0.05	−1.80; 1.70	
Seniority (reference: less than 5 y)			.004
5‐10 y	1.05	0.13; 1.98	
10‐15 y	1.78	0.61; 2.95	
More than 15 y	−0.24	−1.40; 0.92	
More than 50 working hours per week (reference: ≤50 h)	−0.29	−1.07; 0.50	.477
Shift work (reference: no)	−0.55	−1.37; 0.28	.192
History of musculoskeletal diseases (reference: no)	−1.53	−2.74; −0.32	.013
Rural hospital location (reference: urban region)	0.70	−0.12; 1.52	.096
Psychological distress	−0.75	−0.85; −0.66	<.001

^a^ Adjusted for gender.

The multivariate analyses showed that there were associations between quality of life and MMS, being female, being aged between 50 and 60 (reference: 19‐29), and psychological distress. Seniority was removed from the model due to multi‐collinear phenomena with age. Nurses with MS in two to four sites and MS in five sites or more had a lower quality of life score than those who had no MS at any site. For more detail, the unstandardized coefficients of MS in two to four sites and that of five sites or more were −2.22 (95% CI: −3.11 to −1.33) and −3.62 (95% CI: −4.91 to −2.33) respectively. This means that having a higher number of anatomical sites of MS is associated with a worse quality of life. Women (compared to men) and nurses aged between 50 and 60 years (compared to nurses aged between 19 and 29 years) had a lower quality of life score. The quality of life score decreased as the psychological distress score increased. (Table 3).

**TABLE 3:** Multivariate linear regression analysis of quality of life

Independent variable	β	95% CI	*P*
Musculoskeletal symptoms (reference: no site)			<.001
1 site	−0.99	−2.00; 0.02	
2‐4 sites	−2.22	−3.11; −1.33	
5 sites or more	−3.62	−4.91; −2.33	
Female gender (reference: male gender)	−2.34	−3.24; −1.43	<.001
Age (reference: 19‐29)			.004
30‐39	0.13	−0.64; 0.91	
40‐49	−0.70	−1.79; 0.39	
50‐60	−3.11	−4.95; −1.27	
Shift work (reference: no)	−0.43	−1.17; 0.30	.249
History of musculoskeletal diseases (reference: no)	−0.20	−1.34; 0.94	.732
Rural hospital location (reference: urban region)	0.17	−0.56; 0.91	.642
Psychological distress	−0.69	−0.79; −0.59	<.001

Note

Adjusted *R* ^2^ at final model: 0.238.

Collinearity statistics: all of VIF (Variance inflation factor) <2.

Durbin‐Watson: 1.960.

## 4 DISCUSSION

The main results of this study include the following observations:

1. The prevalence of MMS during the past 12 months in men and women was high: 37.6% and 57.1% respectively.
2. Nurses with MMS had a lower quality of life score than those who had no MS at any site.
3. Other factors impair the quality of life for nurses: gender (women), age (50‐60), and psychological distress.

The focus on MMS is a key feature of this study, since there have been few previous studies on MMS in nurses, especially on the factors affecting the appearance of MMS and related issues. A small number of studies of MMS have included nursing in the sample. For example, a survey among 224 nurses, 200 office workers, and 140 postal clerks in Crete, Greece, showed that two thirds of the study sample reported pain in two body sites or more during the previous 12 months, and in 23% of the sample more than three sites were affected.^6^ Another study looking at responses from 1348 health‐care sector employees revealed that over 52% reported pain in multiple body sites and 19% reported pain in one site.^7^ These results show that the MMS prevalence is high and notably higher than those of MS in one site. Several studies on workers in other occupations have produced similar results in the prevalence of MMS: More than 56% of employees in a food processing company reported multisite pain^19^; and 19% of female kitchen workers reported pain in two body sites, and 53% in at least three sites, during the previous three months.^20^ From these data, it is reasonable to conclude that the prevalence of MMS in the general working population is high, something underlined by Parot's study, in which two thirds of workers reported MS in more than one anatomical site.^8^ However, it is difficult to compare these results because the studies contain many differences in terms of subjects, the definition of selection, MMS evaluation criteria (at least two or three anatomical sites), and also language and culture. The results of this study will therefore form both the basis and the premise for further research on nurses.

The results in the final model reveal several factors that damage the quality of life for nurses: MS at two sites or more (MMS), gender (women), age (50‐60), and psychological distress. When discussing the relationship between quality of life, MMS, and psychological distress, the greater the number of sites and the higher the level of psychological distress, the greater the decrease in quality of life. As noted above, few studies have looked at the relationship between MMS and nurses' quality of life, although several studies have highlighted the impact of MSDs in general on nurses' quality of life. A study in Iran has shown that MSDs negatively affect almost all aspects of quality of life for nurses, especially in terms of physical function.^21^ A British study by Joslin concluded that nurses currently suffering neck pain had significantly poorer mental health, physical health, and overall quality of life score (evaluated by SF‐36 questionnaire).^22^ Looking at other target populations, MMS has been shown to negatively impact study participants in many ways. The most typical of these is a reduction in the ability to work, or the danger of such a reduction when the subject is suffering from MS in many sites.^23^ A study by Neupane et al among employees in the health‐care sector pointed to an association between multisite musculoskeletal pain, poor work‐life balance, and physical and psychosocial hazard variables.^7^ Solidaki et al concluded that pain at multiple anatomical sites was common and strongly associated with somatization, which may have a more important influence on multisite pain than pain limited to a single anatomical site.^6^ A study by Sembajwe revealed significant associations between psychosocial demands and multisite musculoskeletal pain among patient care associates, nurses, and administrative personnel, both male and female.^10^ In a 2008 study, most people with musculoskeletal pain reported pain from a number of sites, while localized pain (single site pain) was relatively rare, and musculoskeletal pain at multiple sites had a large impact on physical, mental, and daily and social activities.^24^ Using both SF‐36 and EQ‐5D questionnaires, Picavet et al found that people with multiple musculoskeletal diseases had the poorest health‐related quality of life (which had the effect of aggravating mental health conditions, anxiety, and depression) when compared to those either without or with only one disease.^25^ From the evidence above, it can thus be seen that MMS has many negative effects on workers in terms of both physical and mental health, so the quality of life will decrease in turn. This is confirmed by the results of this study.

When considering the relationship between mental issues and quality of life in nurses, the results from this study show that the higher the level of psychological distress, the more likely it is that quality of life will be affected. Orenius et al concluded that anxiety predicted a significant negative change in health‐related quality of life among patients with chronic musculoskeletal pain.^26^ Mathiesen suggested that, in the general population, psychological distress has a significant impact on quality of life,^27^ while the same is true across different population groups.^28^, ^29^

Regarding age, quality of life for ages 50‐60 is worse than for ages 19‐29. The 50‐60 age range is the final one before retirement in Vietnam, and certain aspects of physical health begin to decline as the body ages. Serious illness is more common in this age group. Although subjects may have achieved financial stability at this age, only one question was related to financial issues in the study's 14 questions about the quality of life. Many studies have shown that quality of life is poor in the elderly compared to the young. A recent survey of 15 386 people across 13 European countries, Canada, and the USA concluded that the age group 50‐59 has the lowest quality of life indicators, with the 18‐29 age group having the highest, in both men and women.^30^ Several Asian studies on the general population have also shown that health‐related quality of life reduces with increasing age.^31^

In this study, the Q‐LES‐Q‐SF questionnaire was used to assess respondents' quality of life. The Q‐LES‐Q and its short form (Q‐LES‐Q‐SF) are among the most frequently used outcome measures in psychiatric research, especially in studies of patients with mental health problems (such as mental disorders, mood disorders, and depression) to measure the degree of enjoyment and satisfaction experienced by subjects in various areas of daily functioning.^16^, ^32^ However, the questionnaires have also been used with a sample of non‐patients in the community and found to have good test‐retest reliability.^32^ This is another reason why this questionnaire was used in this research. Almost all previous studies on quality of life for nurses in particular, and for other subjects in general, have used SF‐36,^33^ EQ‐5D,^34^ or WHO quality of life (WHOQOL) questionnaires.^35^ Although all have been used in Vietnam (the SF‐36 was translated to Vietnamese and evaluated in the Vietnamese population^36^; the Vietnamese version of the EQ‐5D was provided by the EuroQoL and culturally adapted in Vietnam^37^; and the WHOQOL has also been translated into Vietnamese and used in several studies^38^), they are all long and complicated to use. In contrast, the Q‐LES‐Q‐SF questionnaire is short (containing just 14 questions), easy to use, and has been validated in a Vietnamese context.^17^ Along with the fact that no previous study has used this questionnaire on nurses, the aforementioned reasons constitute the grounds for choosing the Q‐LES‐Q‐SF for this study. The K6 questionnaire to measure psychological distress is similarly very concise, containing only six questions, being easy to use, and having been standardized into the Vietnamese language and used in research in Vietnam.^39^

This was a cross‐sectional descriptive study, so it was not possible to specify the causal relationship between MMS, psychological distress, quality of life, and other potential variables. It was only the intention of this study to establish whether they were related to each other. When discussing the research power, its influencing factors include the significance criterion (or alpha), effect size, and sample size. Power is increased when a researcher increases sample size, as well as when a researcher increases effect sizes and significance levels. In this study, the alpha fixed at 0.05, the sample size was not previously estimated. Besides, the main goal is to find the relationship between quality of life and MMS, so the effect size, equals 0.092, was calculated in the case of ANOVA test, and fell in the middle of the medium (0.06) and large effect (0.14).^40^ Therefore, although this study did not estimate the prior sample size, the statistical power level is sufficient and can be acceptable.

Although there were some limitations (it was a cross‐sectional descriptive study, there was recall information bias and subjective judgment of participants when answering questions, and clinical diagnostic criteria for MSDs did not apply), this is the first study in Vietnam on this topic among nurses, with the research sample highly representative of all nurses in Haiphong in particular and in Vietnam in general.

## 5 CONCLUSION

This study clarifies the high prevalence of MMS and further factors impairing the quality of life for nurses, such as gender (female), age (50‐60 years old), psychological distress, and MMS. Further in‐depth studies are needed to deal with the causal relationship between these indicators.

## DISCLOSURE

*Approval of the research protocol*: The study was approved by the Institutional Review Board of the Haiphong University of Medicine and Pharmacy and authorized by the Haiphong Department of Health to implement the study at its district hospitals. *Informed consent*: All study participants provided informed consent before recruitment in the survey. *Registry and the registration no. of the study/trial*: N/A. *Animal studies*: N/A. *Conflict of interest*: The authors declare no conflicts of interest regarding the publication of this paper.

## AUTHOR CONTRIBUTIONS

All listed authors have significantly contributed to this work. Nguyen Thanh Hai, Hoang Duc Luan, Hoang Thi Giang, Nguyen Van Khai, and Pham Minh Khue substantially worked on the conception, design, and process of the study. Jean‐Dominique Dewitte and Yves Roquelaure guided the study and contributed to the acquisition and interpretation of data. Nguyen Thanh Hai and Julie Bodin substantially contributed to the analysis and interpretation of data and writing of the manuscript. All authors worked on revising the manuscript for intellectual content and final approval of the version to be published.

## Data Availability

The SPSS data used to support the findings of this study are available from the corresponding author upon request.
